# Prognostic analysis of Behçet’s disease with aortic regurgitation or involvement

**DOI:** 10.1007/s12471-021-01567-6

**Published:** 2021-04-20

**Authors:** X. Li, X. Wen, J. Xu, Q. Lin, L. Liu

**Affiliations:** 1 Department of Cardiovascular Medicine, The Second Xiangya Hospital, Central South University, Changsha, Hunan China; 2 Research Institute of Blood Lipid and Atherosclerosis, Central South University, Changsha, Hunan China; 3 Modern Cardiovascular Disease Clinical Technology Research Centre of Hunan Province, Changsha, Hunan China; 4 Cardiovascular Disease Research Centre of Hunan Province, Changsha, Hunan China; 5 Xiangya School of Medicine, Central South University, Changsha, Hunan China

**Keywords:** Behçet’s disease, Aortic regurgitation, Aortic involvement, Diagnosis, Prognosis

## Abstract

**Background:**

Aortic regurgitation is the most common cardiovascular damage in Chinese patients with Behçet’s disease (BD) and is usually associated with aortic disease. These patients are easily misdiagnosed, and their prognosis is poor, even after surgical treatment. This study aimed to analyse potential factors that can improve the prognosis of BD patients with aortic regurgitation and/or aortic involvement.

**Methods:**

Twenty-two patients with diagnosed or suspected BD as well as aortic regurgitation and/or aortic involvement in our hospital from 2012 through 2017 were collected in this study. Their clinical characteristics were listed, and the diagnosis of BD was evaluated by two different criteria sets. The influences of surgical treatment and immunosuppressive therapy (IST) on their prognosis were also explored.

**Results:**

The diagnostic positive rate of the International Criteria for Behçet’s Disease was higher than that of the International Study Group criteria (kappa value 0.31, *p* < 0.05), indicating that the diagnostic consistency between the criteria sets was poor. There was no significant difference in survival between patients who had undergone ≤ 1 operation and those with ≥ 2 operations. Aortic valve replacement alone or in combination with aortic root replacement had no significant effect on the incidence of reoperation or death, but IST did significantly reduce this incidence (*p* < 0.05). However, there was no significant difference in the occurrence of reoperation or death between preoperative and postoperative IST versus postoperative IST only.

**Conclusion:**

IST significantly improved the prognosis of BD patients with aortic regurgitation and/or aortic involvement.

**Supplementary Information:**

The online version of this article (10.1007/s12471-021-01567-6) contains supplementary material, which is available to authorized users.

## What’s new?

- We found that in Behçet’s disease (BD) patients with aortic regurgitation and/or aortic involvement, the diagnostic positive rate of the International Criteria for Behçet’s Disease was higher than that of the International Study Group criteria.
- Immunosuppressive therapy significantly improved the prognosis of BD patients with aortic regurgitation and/or aortic involvement.

## Introduction

Behçet’s disease (BD) is a chronic systemic inflammatory disorder of unknown cause that can affect the cardiovascular system, where it manifests as valve dysfunction and vascular damage [1]. Aortic regurgitation is the most common cardiovascular damage in Chinese BD patients and is usually associated with aortic disease [2]. While the diagnosis of BD is usually based on the clinical manifestations of noncardiovascular systems [3], the prognosis of BD patients is often determined by the cardiovascular complications [4, 5]. BD patients with cardiovascular complications can initially visit the Department of Cardiovascular Medicine or Surgery [6]. Because the noncardiovascular manifestations are often slight, these patients can be misdiagnosed and have to go through several rounds of surgery due to various postoperative complications, resulting in a very poor prognosis [5, 7, 8].

Cases of BD with cardiovascular damage are commonly reported in countries related to the former Silk Road, especially in East Asian countries [9]; there are relatively few similar cases in China [10]. Recently, cardiovascular doctors have realised that patients with unexplained aortic regurgitation and/or vascular fistula after related operations should be regarded as having possible BD [11].

To study ways to improve the prognosis of BD with cardiovascular involvement, we collected and analysed all cases with diagnosed or suspected BD as well as aortic regurgitation and/or aortic involvement in our hospital in China from 2012–2017.

## Methods

### Subjects

**Fig. 1:**
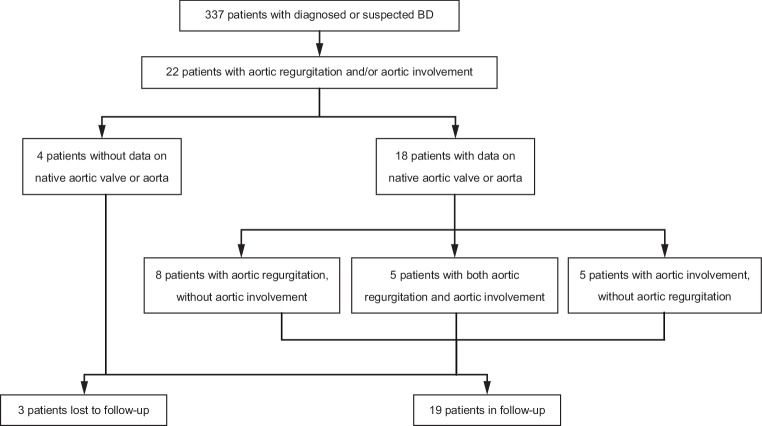
Flow chart of study patients. *BD* Behçet’s disease

We reviewed medical records of 337 patients who had been diagnosed with or suspected of BD in The Second Xiangya Hospital, Central South University in Changsha, China in the period 2012–2017, 22 of whom also had aortic regurgitation and/or aortic involvement (Fig. 1). Clinical manifestations, preoperative echocardiographic findings and operative information of all patients were collected and retrospectively analysed. The study was approved by the Ethics Committee of The Second Xiangya Hospital, and informed consent was obtained from all participants.

There are two sets of diagnostic criteria for BD. The first are the criteria of the International Study Group (ISG). The major clinical features include oral and genital ulceration, ocular lesions, skin lesions and positive pathergy test results. Patients with recurrent oral ulceration and at least two of the described other features are diagnosed with definite BD [3]. The second set is called the International Criteria for Behçet’s Disease (ICBD), where two points each are assigned to oral ulceration, genital ulceration or ocular lesions and one point each to skin lesions, vascular manifestations or neurological manifestations. A pathergy test is optional, but if is conducted, one extra point may be assigned for a positive result [12]. Patients with four points are diagnosed as having BD.

In our study population, once BD was diagnosed or suspected, rheumatologists were consulted in case of diagnostic problems and for medical management. Immunosuppressive treatments (e.g. thalidomide, azathioprine, cyclophosphamide or methotrexate), including corticosteroids(e.g. prednisolone or methylprednisolone), were administered preoperatively and postoperatively, although there was no standard immunosuppressive therapy (IST) regimen for BD patients with cardiovascular involvement at the time. The operation indication, method and timing were based on consensus between cardiovascular surgeons and rheumatic immunologists.

### Statistical analysis

Categorical variables are expressed as number (percentage) and continuous variables as mean ± standard deviation, as appropriate. An unpaired Student’s *t*-test and a chi-squared test were used to analyse quantitative and qualitative variables, respectively. Kappa value was used to measure the agreement between the ISG criteria and the ICBD in BD patients with cardiovascular involvement. Kaplan-Meier curves were generated to estimate the survival function of time to events according to different treatments. Differences in the curves were compared using the log-rank test.

All statistical analyses were conducted using SPSS 22.0, GraphPad Prism 7.0 software and R software version 3.6.1. All *p*-values were two-tailed, and *p* < 0.05 was considered to be statistically significant.

## Results

### General characteristics

In total, 22 patients who had been diagnosed as having definite or suspected BD with aortic regurgitation and/or aortic involvement were included in this study, of whom 15 men (68.2%) and 7 women (31.8%). Their mean age was 41.6 ± 9.9 years (range 23–60) when they were found with aortic regurgitation and/or aortic involvement.

### Extracardiac characteristics and diagnosis of BD

Among the 22 patients, the incidence rate of oral ulceration, genital ulceration and skin lesion was 95.5%, 81.8% and 45.5%, respectively. Eight patients (36.4%) were examined by an ophthalmologist, whereas only 2 of whom had an ocular lesion. Of the 12 patients (54.5%) who had undergone a pathergy test, 3 had a positive test result and 1 had a suspicious positive result.

Throughout the course of the disease, 10 patients were diagnosed with BD according to the ISG criteria; the remaining 12 patients were suspected of having BD. Using the ICBD, 18 patients were diagnosed with BD, although 8 of them did not meet the ISG criteria; the remaining 4 patients had 2 or 3 points and were suspected of having BD (see Tab. 1 in Electronic Supplementary Material). The kappa value was 0.31 (*p* < 0.05), which indicated poor coincidence between the two diagnostic criteria sets. The diagnostic positive rate of the ICBD (81.8%) was higher than that of the ISG criteria (45.5%) in BD patients with aortic regurgitation and/or aortic involvement.

### Cardiovascular characteristics

Three patients had aortic regurgitation and 1 patient showed aortic involvement after their last operation at other hospitals. The initial information on their native valve and aorta before their first operation was not available. For, the other 18 patients, an abnormal native valve or aorta was detected by echocardiography or computed tomography angiography at our hospital.

Among these 18 patients, 13 (72.2%) had aortic regurgitation. Of these 13 patients, 8 (61.5%) had an aortic valve prolapse, 4 (30.8%) a bicuspid aortic valve, 2 (15.4%) an aortic valve stenosis, 1 (7.7%) had a paravalvular abscess and 1 (7.7%) an aortic valve vegetation.

**Fig. 2:**
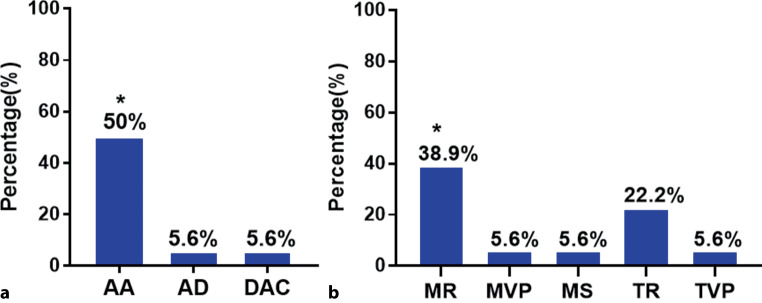
Constituent ratio of different types of aortic or atrioventricular valve involvement in patients with definite or suspected Behçet’s disease (*BD*). **a** Aortic involvement consisted of aortic aneurysm (*AA*), aortic dissection (*AD*) and descending aortic constriction (*DAC*). **b** Atrioventricular valve involvement consisted of mitral regurgitation (*MR*), mitral valve prolapse (*MVP*), mitral stenosis (*MS*), tricuspid regurgitation (*TR*) and tricuspid valve prolapse (*TVP*). *P < 0.05

Among the 18 patients, 9 (50%) had an aortic aneurysm, 1 (5.6%) an aortic dissection and 1 (5.6%) a descending aortic constriction (Fig. 2a). In the 9 patients with an aortic aneurysm, the mean aortic diameter was 5.4 ± 1.6 cm.

Atrioventricular valve involvement in the 18 patients was usually mild and consisted of mitral regurgitation in 7 patients (38.9%), mitral valve prolapse in 1 (5.6%), mitral stenosis in 1 (5.6%), tricuspid regurgitation in 4 (22.2%) and tricuspid valve prolapse in 1 (5.6%) (Fig. 2b).

### Operational complications and outcomes

Of the 22 patients, 3 were lost after discharge and their data were not used in the following statistical analysis; 19 patients were followed-up from discharge to September 2019. Of these 19 patients, 8 never received a cardiovascular operation and 11 were operated 22 times, with an average of 2 times per patient.

Among the 11 operated patients, 6 underwent aortic valve replacement at the first operation. Because of postoperative complications, 3 received aortic valve replacement at the second operation and 2 underwent a Bentall procedure at the third operation. Three of the 11 patients received aortic surgery at the first operation, 2 of whom received interventional treatment of the aortic aneurysm only; 1 received aortic root replacement at the first operation and underwent four more operations because of postoperative complications. One of the 11 patients received both aortic valve and aortic root replacement at the first operation; he later had two more aortic fistula repairs. Another patient received aortic root replacement once after discharge from our hospital.

Of the 19 patients, 15 survived, while 4 died during follow-up. One patient died at the third day after the third open thoracotomy operation (Bentall operation) because of both respiratory and circulatory failure. The second and third patient were subjected to one round of aortic valve replacement, but one patient died from paravalvular leakage after 19 months, and the other died from the same cause in combination with prosthetic valve endocarditis after 7 months. The fourth patient did not undergo any operation and died of an aneurysm rupture (see Tab. 2 in Electronic Supplementary Material).

**Fig. 3:**
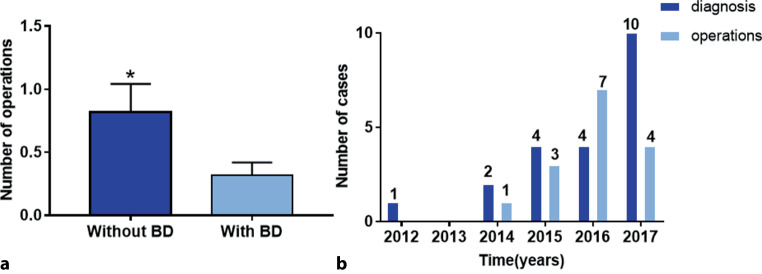
Impact of Behçet’s disease (*BD*) diagnosis or suspicion on choice of operation. **a** Number of operations performed per patient with and without diagnosis or suspicion of BD. *P < 0.05 when compared with number of operations after diagnosis or suspicion of BD. **b** Number of patients with definite or suspected BD and number of operations in these patients from 2012–2017

Among the 11 operated patients, the number of operations before and after the diagnosis or suspicion of BD was compared. Once BD was diagnosed or suspected clinically, the number of operations was significantly lower (*p* < 0.05, Fig. 3a). There was a tendency of an increase in the number of cases with a diagnosis or suspicion of BD from 2012–2017, but the number of operations decreased in 2017 compared with 2016 (Fig. 3b).

**Fig. 4:**
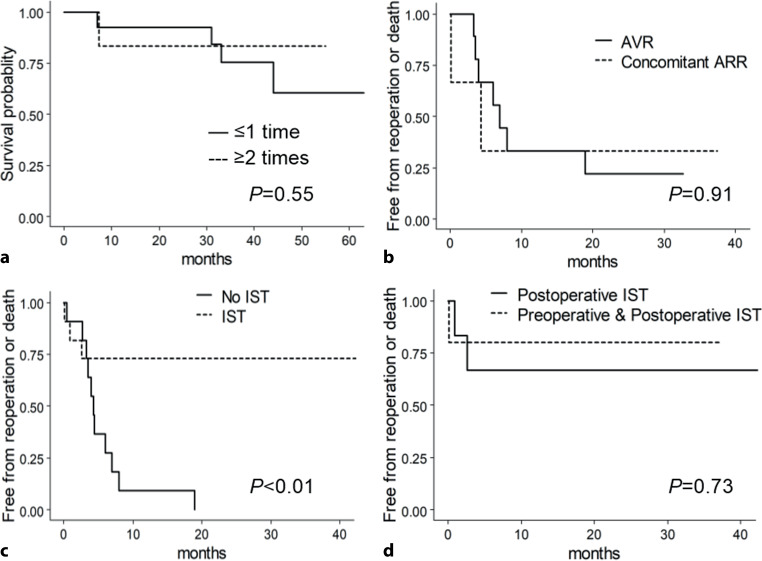
Effects of operation or immunosuppressive therapy (*IST*) on prognosis. **a** Effect of number of operations on survival. **b** Kaplan-Meier curve showing effect of operation method on incidence of reoperation or death. Operation methods were aortic valve replacement (*AVR*) and AVR in combination with aortic root replacement (i.e. concomitant aortic root replacement). **c** Kaplan-Meier curve showing effect of perioperative IST on incidence of reoperation or death. **d** Kaplan-Meier curve showing effect of preoperative and postoperative IST or postoperative IST only on incidence of reoperation or death

Survival curve analysis showed there was no difference in survival between patients with ≤ 1 operation and those with ≥ 2 operations (Fig. 4a). In 7 patients with aortic valve replacement, reoperation or death occurred after seven simple aortic valve replacement surgeries and after two concomitant aortic root replacements. Kaplan-Meier curve analysis showed there was no difference in freedom from reoperation or death, regardless of choosing aortic valve replacement or aortic valve with aortic root replacement for patients with aortic regurgitation (Fig. 4b).

### Immunosuppressive therapy and outcomes

Among the 19 patients, 11 patients underwent 22 operations in total, ranging from 1–5 per patient. Five patients (5 operations) received preoperative and postoperative IST and 6 patients (6 operations) received postoperative IST only; 7 patients (11 operations) did not receive any IST.

Kaplan-Meier curve analysis showed that patients receiving perioperative IST had a significantly lower incidence of reoperation or dead than those without IST (Fig. 4c). However, there was no significant difference in freedom from reoperation or death between preoperative plus postoperative IST and postoperative IST only (Fig. 4d).

## Discussion

Research has shown that the ICBD have a higher positive rate than the ISG criteria in BD patients with cardiovascular involvement [13]. It was reported that in many cases, BD patients with severe aortic regurgitation or other vascular involvement do not fully meet the ISG criteria or the ICBD [5, 13]. Indeed, in our study, 4 patients were suspected of having BD according the ICBD. All of them received IST for the clinical suspicion of BD and survived during the follow-up, regardless of whether they underwent surgery. Moreover, due to the rarity of BD cases with severe cardiovascular involvement and small sample sizes, statistical analysis was carried out on the pool of confirmed and suspected BD patients in our study, as well as in similar studies [13, 14].

The most common manifestation of endocardiac involvement in BD is aortic regurgitation [5, 11, 13]. In this study, the main manifestation of aortic involvement was aneurysmal dilation. Some scholars have proposed that aortic regurgitation can be secondary to aortic dilation [15]. Others have suggested that BD patients can have both aortic dilation and valve damage simultaneously due to BD [7, 16], because the incidence of aortic regurgitation in BD is higher than that of aortic involvement, which is consistent with the finding of this study. Several authors have recommended that clinicians immediately consider the possibility of BD when they observe aortic regurgitation with unknown cause in young subjects or recurrent aortic regurgitation after operation [8, 13, 14]. The ultrasonic manifestations of BD-related AR include echo-free spaces and vegetation-like lesions [7, 8], which are easily misdiagnosed as myocardial abscess or bacterial vegetations [11]. Some patients can be misdiagnosed with infective endocarditis and subsequently receive antibiotic treatment or valve replacement [7, 8, 17]. Thus, BD should be excluded in patients with AR and/or aortic aneurysm who are suspected of having BD.

For BD patients with cardiovascular involvement, surgery may be life-saving, but could also have disastrous consequences. The mechanism of paravalvular leakage and valve detachment after aortic valve replacement may be related to vasculitis, tissue fragility and high suture tension, among others [18]. Aortic valve replacement can connect the artificial valve to the aortic wall with vasculitis and normal appearance, which may lead to complications. The recurrence rate of complications after reoperation is very high. Repeated aortic valve replacement can neither effectively prevent the occurrence of complications [5], nor improve the prognosis of those specific patients [7]. Therefore, reducing the number of surgical complications in BD patients has become the focus for cardiovascular specialists.

Compared with aortic valve replacement alone, combining aortic valve and aortic root replacement leads to significantly fewer complications [10, 19, 20]. This combined operation is suitable for patients with severe aortic regurgitation with aortic involvement. Two types of combined aortic root and aortic valve replacement were used in the current study: Bentall operation and Wheat operation. Japanese researchers have found that the incidence of valve detachment is lower in patients who underwent a translocated Bentall procedure or valved conduit procedures (a modified Bentall operation) than those who had aortic valve replacement procedures [20, 21].

However, recent evidence has shown that the overall differences in event-free survival between patients who underwent aortic root replacement and those in the isolated aortic valve replacement group are insignificant [22]. Due to the small sample size and the heterogeneity of subjects in our study, the influence of the operation number or method on the prognosis cannot be determined. It can be conjectured that if there is no effective treatment for BD patients, those with aortic regurgitation may fall into a vicious circle of repeated complications when they repeatedly receive simple aortic valve replacement.

Timely BD diagnosis or even suspicion of BD can change clinicians’ choice of treatment. Because of the high incidence of complications in patients with BD after cardiovascular surgery, once clinicians begin to suspect BD or make a diagnosis of this disease, surgical operation is rarely considered as the next treatment [8]. We observed a decrease in operation number and an increase in number of diagnosed cases in 2017. This indicated that the surgeons recently had enough understanding and a certain vigilance regarding the cardiovascular characteristics of BD.

For BD patients with cardiovascular involvement, the most important treatment is not simply to improve the technology of cardiovascular surgery, but to actively treat and control inflammation related to BD by immunosuppressive agents [20]. According to the recommendations for the management of Behçet’s syndrome (i.e. BD), updated in 2018, IST significantly improves the prognosis of BD patients with artery involvement [23]. However, the effect of IST on the prognosis of BD patients with aortic regurgitation is not mentioned in these recommendations [23], although Korean scholars had reported the definite effect of IST on the prognosis of 9 BD patients with aortic regurgitation in 2002 [5] and 19 cases in 2009 [7].

Research on the impact of IST on the prognosis of BD patients with aortic regurgitation or involvement is extremely rare in China, which was an important reason for us to carry out this study. More importantly, the progress in IST has affected the treatment options for Chinese doctors. They have tried to avoid surgery in those patients with diagnosed or even suspected BD as much as possible and switched to other treatments, such as IST. Interestingly, postoperative IST significantly reduces postoperative complications after aortic valve replacement in patients with BD [5], while short-term preoperative IST has no significant effect on the occurrence of postoperative paravalvular leakage [13].

Pathological examination has shown that short-term preoperative IST can control the acute inflammatory response to a certain extent, but that there is still focal chronic inflammation. In any event, long-term IST is particularly important to reduce mortality and postoperative complications. Our study supports the notion that Chinese BD patients with aortic regurgitation and/or aortic involvement can benefit from IST, especially from long-term postoperative IST.

### Study limitations

This study has several limitations. Firstly, the sample size was small. Because cardiovascular involvement is rare in BD patients, only 22 such patients were included, making the study susceptible to low statistical power.

Secondly, because of the retrospective study design and the lack of a standard IST regimen for BD patients with cardiovascular involvement, the individual IST was administrated by rheumatologists according to the types and severity of organ involvement. Treatment heterogeneity made it difficult to compare the different IST regimens.

Thirdly, the operation indications were different. The operations were conducted in patients with intractable heart failure due to aortic regurgitation, aortic aneurysm with high risk of rupture or aortic dissection. Once those patients are diagnosed with definite or suspected BD, surgery should be avoided or delayed if they are not in a life-threatening situation.

## Conclusion

In this study, IST significantly improved the prognosis of BD patients with aortic regurgitation and/or aortic involvement.

## Supplementary Information

Table 1. Extracardiovascular manifestations in all patients

Table 2. The records of operations and immunosuppressive therapies of all patients
