# Supplementary material for: Prognostic analysis of Behçet’s disease with aortic regurgitation or involvement
Source: Neth Heart J. 2021 Apr 20;30(3):172–80. doi: 10.1007/s12471-021-01567-6 (PMC8881513; doi:10.1007/s12471-021-01567-6)
Supplement: Supplementary file 1 — Table 1. Extracardiovascular manifestations in all patients [file 12471_2021_1567_MOESM1_ESM.docx]

Table 1. Extracardiovascular manifestations in all patients

| Patient | Sex, age | Oral ulceration | Genital ulceration | Ocular lesion | Skin lesion | Pathergy test | Additional symptoms | ISG criteria | ICBD  criteria |
| --- | --- | --- | --- | --- | --- | --- | --- | --- | --- |
| 1 | M,44 | + | + | - | + | + | AAA | A | 7 |
| 2 | F,47 | + | + | NA | + | - |  | A | 5 |
| 3 | M,50 | + | + | NA | + | NA |  | A | 5 |
| 4 | F,52 | + | + | + | + | NA |  | A | 7 |
| 5 | M,45 | + | + | NA | + | NA |  | A | 5 |
| 6 | F,46 | + | + | NA | - | NA |  | D | 4 |
| 7 | F,55 | + | + | + | - | + | AA | A | 8 |
| 8 | M,42 | + | + | NA | + | S | AA | A | 6 |
| 9 | M,27 | + | + | NA | + | + | AD | A | 7 |
| 10 | M,32 | + | + | NA | - | NA | AA | D | 5 |
| 11 | M,37 | - | - | - | + | - | AAA | D | 2 |
| 12 | M,35 | + | + | NA | - | NA |  | D | 4 |
| 13 | M,23 | + | + | - | - | - | AA | D | 5 |
| 14 | M,41 | + | + | - | + | - | Peptic ulcer | A | 5 |
| 15 | F,27 | + | + | NA | + | - | Stenosis of DA | A | 6 |
| 16 | F,44 | + | + | NA | - | NA |  | D | 4 |
| 17 | M,28 | + | - | - | + | - |  | D | 3 |
| 18 | M,36 | + | - | NA | - | - |  | D | 2 |
| 19 | M,45 | + | - | - | + | - |  | D | 3 |
| 20 | M,60 | + | + | NA | - | NA | AA | D | 5 |
| 21 | F,48 | + | + | NA | - | NA | AA | D | 5 |
| 22 | M,51 | + | + | NA | - | NA |  | D | 4 |

Note:

ISG: International Study Group; ICBD: International Criteria for Behcet’s Disease; M: male; +：positive；-：negative；AAA: Abdominal aortic aneurysm; A: accepted; F: female; NA: not available; D: denied; S: suspected; AA: aortic aneurysm; DA: descending aorta; PAH: pulmonary arterial hypertension.
