# Supplementary material for: Prognostic analysis of Behçet’s disease with aortic regurgitation or involvement
Source: Neth Heart J. 2021 Apr 20;30(3):172–80. doi: 10.1007/s12471-021-01567-6 (PMC8881513; doi:10.1007/s12471-021-01567-6)
Supplement: Supplementary file 2 — Table 2. The records of operations and immunosuppressive therapies of all patients [file 12471_2021_1567_MOESM2_ESM.docx]

Table 2. The records of operations and immunosuppressive therapies of all patients

| Patient | Preoperative diagnosis | First operation | Time at diagnose | IST | Times of operation | Complication  (interval) | outcome |
| --- | --- | --- | --- | --- | --- | --- | --- |
| 1 | AAA | EVAR | 5m after 1^st^ operation | T, MP | 1 | PSA(3m) | Loss |
| 2 | BD,AR | - | 6y before 1^st^ operation | T,P | 0 | - | S |
| 3 | RHD | AVR | Immediately before 2^nd^ operation | T | 1 | PVL (5m) | D |
| 4 | BD,AR | - | 3y before 1^st^ operation | T | 0 | - | S |
| 5 | AR | AVR | 2m before 3^rd^ operation | T,P | 3(AVR, Bentall) | PVL(3m, 1m) | D |
| 6 | Suspected BD | - | Immediately before 1^st^ operation | - | 0 | - | S |
| 7 | BD,AR | - | Immediately before 1^st^ operation | T,P,MTX | 0 | - | S |
| 8 | BD | - | 19m before 1^st^ operation | T,P,MTX | 0 | - | D |
| 9 | AD | Sun’s procedure | 0.5m before 5^th^ operation | MP | 5(Repair of anastomotic fistula) | Anastomosis fistula(2m) | S |
| 10 | TAA | EVAR | 0.5m after 1^st^ operation | T,MP,CTX | 1 | PSA(20d) | S |
| 11 | AAA | TSGP | 2m after 1^st^ operation | - | 1 | - | S |
| 12 | CAI | AVR | 2m before 3^rd^ operation | T | 3(AVR, Bentall) | PVL, | S |
| 13 | MFS | Wheat | 2m after 2^nd^ operation | T,C | 3(Repair of anastomotic fistula) | Anastomotic fistula(4m,2m) | S |
| 14 | BD, AR | - | 7y before 1^st^ operation | T,P | 0 | - | Loss |
| 15 | Arteritis | - | Immediately before 1^st^ operation | MP ,CTX, T | 0 | - | S |
| 16 | BD | - | Immediately before 1^st^ operation | MP , T | 0 | - | Loss |
| 17 | VHD | AVR | 1m after 2^nd^ operation | MP,IVIG,CTX | 2(AVR) | PVL,PVA (1m,0.7m) | S |
| 18 | VHD | AVR | Immediately after 1^st^ operation | MP | 1 | - | S |
| 19 | BD, AR | - | Immediately before 1^st^ operation | T | 0 | - | S |
| 20 | AA | ARR | 3y before 1^st^ operation | MP, T, C | 1 | - | S |
| 21 | AA | - | 10y before 1^st^ operation | MP,CTX | 0 | - | D |
| 22 | AR | AVR | 7m after 1^st^ operation | - | 1 | PVL (6m) | D |

Note:

IST：immunosuppressive therapy; AAA: abdominal aortic aneurysm; EVAR: endovascular aorta repair; T: thalidomide; MP: methylprednisolone; PSA: pseudoaneurysm; BD: Behcet’s disease; AR: aortic regurgitation; P: prednisolone; S : survival; RHD: rheumatic heart disease; AVR: aortic valve replacement; PVL: paravalvular leakage; D: death; MTX: methotrexate; AD: aortic dissection; TAA: thoracic aortic aneurysm; CTX: cyclophosphamide; TSGP: transluminal stent-graft placement; CAI: congenital aortic insufficiency; MFS: Marfan syndrome; C: colchicines; VHD: valvular heart disease; IVIG: intravenous immunoglobulin; PVA: paravalvular abscess; AA: aortic aneurysm; ARR: aortic root replacement.
